# Nutritional Therapy Strategies in Pediatric Crohn’s Disease

**DOI:** 10.3390/nu13010212

**Published:** 2021-01-13

**Authors:** Charlotte M. Verburgt, Mohammed Ghiboub, Marc A. Benninga, Wouter J. de Jonge, Johan E. Van Limbergen

**Affiliations:** 1 Department of Pediatric Gastroenterology and Nutrition, Emma Children’s Hospital, Amsterdam University Medical Centers, 1105 AZ Amsterdam, The Netherlands; c.m.verburgt@amsterdamumc.nl (C.M.V.); m.ghiboub@amsterdamumc.nl (M.G.); m.a.benninga@amsterdamumc.nl (M.A.B.); 2 Tytgat Institute for Liver and Intestinal Research, Amsterdam Gastroenterology Endocrinology Metabolism, Amsterdam University Medical Centers, University of Amsterdam, 1105 BK Amsterdam, The Netherlands; w.j.dejonge@amsterdamumc.nl; 3 Department of Surgery, University of Bonn, 53127 Bonn, Germany; 4 Department of Pediatrics, Dalhousie University, Halifax, NS B3K 6R8, Canada

**Keywords:** pediatric Crohn’s disease, diet, nutritional therapy, inflammation, microbiota

## Abstract

The increase in incidences of pediatric Crohn’s Disease (CD) worldwide has been strongly linked with dietary shifts towards a Westernized diet, ultimately leading to altered gut microbiota and disturbance in intestinal immunity and the metabolome. Multiple clinical studies in children with CD have demonstrated the high efficacy of nutritional therapy with exclusive enteral nutrition (EEN) to induce remission with an excellent safety profile. However, EEN is poorly tolerated, limiting its compliance and clinical application. This has spiked an interest in the development of alternative and better-tolerated nutritional therapy strategies. Several nutritional therapies have now been designed not only to treat the nutritional deficiencies seen in children with active CD but also to correct dysbiosis and reduce intestinal inflammation. In this review, we report the most recent insights regarding nutritional strategies in children with active CD: EEN, partial enteral nutrition (PEN), Crohn’s disease exclusion diet (CDED), and CD treatment-with-eating diet (CD-TREAT). We describe their setup, efficacy, safety, and (dis)advantages as well as some of their potential mechanisms of action and perspectives. A better understanding of different nutritional therapeutic options and their mechanisms will yield better and safer management strategies for children with CD and may address the barriers and limitations of current strategies in children.

## 1. Introduction 

Crohn’s disease (CD) is a chronic disorder that belongs to the group of inflammatory bowel diseases (IBD); it is characterized by transmural inflammation that can affect any area along the proximal-distal axis of the gastrointestinal tract (GI) [1,2,3]. Symptoms often involve abdominal pain, diarrhea, rectal blood loss, and fatigue, and the disease often leads to weight loss and malnutrition [4]. The incidence of CD is increasing worldwide, and disease-onset can occur at any age [5]. Up to 15% of CD patients are diagnosed before the age of 20 [6,7,8]. The incidence of pediatric CD is still increasing and varies from 2.5 to 11.4 per 100,000, although a recent meta-analysis concluded the incidence in Europe to be between 9–10 per 100,000. Few studies have reported on the prevalence of pediatric IBD, but overall, there is an estimated prevalence of 58/100,000, although the contribution of pediatric cases to the overall IBD burden for society has remained low due to the increasing prevalence of adult-onset disease [5,9,10]. While the etiology of CD may be similar between children and adults, children with CD typically have a more extensive/panenteric phenotype; however, the time of progression to stricturing and penetrating complications is similar [11,12]. As their disease course occurs during periods of growth and development, children are particularly vulnerable, and management strategies need to take growth characteristics into account [13,14]. CD is considered a multifactorial disorder, where genetics, environment, gut microbiota, and the immune system interplay to contribute to disease development [3,15,16]. However, despite the extensive research performed on CD, treatment remains focused on immune suppressive measures and its etiology not fully understood.

CD is characterized by the excessive infiltration of leukocytes into the inflamed mucosa and a high level of secreted proinflammatory cytokines [17,18]. Thus, medication regimens are focused on the use of immunomodulators or -suppressants (such as corticosteroids, methotrexates, thiopurines, and biologicals such antitumor necrosis factor alpha (TNFα)) to dampen immune system activity [9,19]. However, multiple side effects, such as the increased risk of infections and malignancy, are associated with the use of immuno-modulators and -suppressants [9]. In turn, the use of corticosteroids in pediatric CD is associated with growth retardation and reduced bone accrual [9,20,21,22].

Extensive studies have been performed to relate microbiome changes with active disease and/or response to treatment [23,24,25,26,27,28,29]. Indeed, microbiome manipulation by means of antibiotics has shown promise as a therapeutic strategy for treating pediatric CD in a randomized controlled trial (RCT) of azithromycin + metronidazole for luminal CD [9,19,30,31]. Antibiotics are also indicated to help maintain remission to anti-TNF in perianal CD [9,32,33]. Although various immunosuppressants and antibiotics can provide therapeutic benefit in CD, a minority of patients maintain remission after induction of remission without maintenance therapy. In addition, there is a substantial rate of primary nonresponse and loss-of-response to immunosuppressants, which leads to a high, unmet need for novel effective therapies [34,35,36,37].

A major factor in intestinal microbiota composition and ecology is diet. Diet has been found to strongly impact gut microbiota, which has been identified as a crucial player in regulating metabolism and the immune response [38,39,40]. Multiple studies have highlighted the impact of changes in dietary intake and consequences of food industrialization (such as the Western diet, which is rich in fats and carbohydrates) on the gut microbiome (dysbiosis) and on increasing pediatric CD incidence [3,5,26,41,42,43]. This has provided a strong rationale for further investigating nutrition as a potential therapy to induce or maintain remission in pediatric CD. While current medical therapy is mainly directed against inflammation, nutritional therapies can be directed toward the correction of dysbiosis and metabolome as well as to the reduction of inflammation [9]. Recent European guidelines have confirmed the central role of dietary therapy (notably exclusive enteral nutrition (EEN)) in the management of mild-to-moderate CD while emphasizing the need for rigorous clinical studies of novel dietary strategies (including the better-tolerated novel Crohn’s disease exclusion diet (CDED)) [9]. Over the past few years, a number of nutritional therapy strategies have been designed to reduce dietary exposure to foods that might adversely impact the microbiome, the intestinal barrier, and innate immunity [3,9,44]. In this review, we gather the most recent advances regarding different nutritional therapies to induce and maintain remission in pediatric CD. We discuss their therapeutic protocols, efficacy, safety, (dis)advantages, and potential mechanisms.

## 2. Methods

In order to give an overview of existing nutritional therapies in pediatric CD and gather the most relevant advances in research in the field of nutritional therapy, we performed a literature review in Medline (PubMed) using “Crohn’s disease”, “nutritional therapy”, “(partial) enteral nutrition”, “diet”, “mechanism”, and “pediatric” as keywords. Reference lists of existing (systematic) reviews of this topic were searched for additional relevant literature. All included articles were in English. There were no specific in- or exclusion criteria for this narrative review. For the description of the different types of nutritional therapy and their efficacy, we focused on research conducted on children. The most contributing articles were selected and are described in Section 4, with an overview of study characteristics and results shown in Table 1.

## 3. Dietary Inflammatory Potential and Risk of Colitis

The persisting rise in incidences of IBD has gone hand in hand with the Westernization of different continents [24,26]. In particular, the Western diet has been largely studied and linked to an increased inflammatory state in a number of diseases, including IBD [40,45,46]. It includes high amounts of processed foods, red meat, high fat, sugar and additive exposure, and a lack of dietary fiber, fruit, and vegetables [39].

The loss of dietary fiber leads to less small chain fatty acid (SCFA) production and a reduction of the available energy source of gut epithelial cells [47]. This influences not only gut microbial composition but also function and, in turn, can impact host immunity [47]. High animal protein intake has been associated with an increased risk of IBD [48,49]. A high sugar diet has been shown to enhance susceptibility to colitis in mice by reducing SCFA and increasing gut permeability [50]. Food additives have also been found to play a negative role in intestinal inflammation, impairing antibacterial responses and suppressing antimicrobial defense mechanisms. They can promote colitis susceptibility in mice by increasing intestinal permeability and significant thinning of the mucous layer [51]. In addition, a positive correlation between emulsifier consumption and increased IBD incidence was found when studying data from different countries [52,53]. Dietary emulsifiers can increase the ability of CD-associated adherent-invasive *E. coli* to adhere to epithelial cells and promote intestinal inflammation in mice [54]. The intake of a diet high in red meat aggravates the severity of dextran sodium sulfate (DSS)-colitis, translated by higher disease activity and histopathological scores [55].

Altogether, these data suggest that an altered dietary pattern can lead to changes in the microbiota (dysbiosis) and altered gut homeostasis and host immunity by promoting inflammation and increasing susceptibility to colitis [38,40]. This indicates the potential of modulating dietary intake as therapy for IBD. To date, mostly preclinical studies in animal models and cell lines have studied the effect of different dietary components on the host; the data remain to be verified in humans [44,56,57,58,59].

## 4. Different Types of Nutritional Therapy and Their Efficacy

There are different types of nutritional therapy being used and explored in pediatric CD [3,9,44]. They differ in treatment duration, induced outcomes, and nutritional composition [60]. Here, we discuss the four main nutritional therapies, varying from the widely implemented EEN to more novel dietary/nutritional strategies. The main results of the clinical studies using nutritional therapies on children with CD, which are described in this section, are summarized in Table 1.

### 4.1. Exclusive Enteral Nutrition (EEN)

EEN consists of a complete liquid formula diet that contains all nutritional requirements and excludes all regular table foods for a determined period of time [61,62]. The main types of EEN formula available are elemental, semielemental, and polymeric, which can be adapted for specific conditions like IBD [63,64,65]. The formulas differ in composition, size, and structure of proteins and fats [65,66]. The daily amount of EEN is based on the estimated energy requirement of an individual [67]. EEN can be administered orally (in case of a polymeric formula) or by a nasogastric tube in case of an inability to meet required daily intake (or to comply with payer-requirements in selected health care systems, where it is stipulated that medical nutrition is only reimbursed when it is delivered through a tube). The exact duration of EEN therapy for induction of remission varies from 6–8 weeks mostly, but there have been reports on the use of EEN for 4–12 weeks [9,61]. After the strict exclusion of solid food during EEN therapy, solid food is gradually reintroduced until a normal intake is reached [9,61].

EEN has been widely accepted as first-line therapy for induction of remission in mild-to-moderate pediatric CD [9]. Multiple studies and meta-analyses of data in children with CD have clearly shown that EEN leads to similar or even superior efficacy in induction of remission compared to corticosteroids [68,69,70]. More importantly, studies have shown that EEN may also be associated with superior mucosal healing and normal CRP remission [57,71,72], which, in turn, are associated with fewer subsequent complications [73]. In general, EEN induces remission in approximately 75–85% of children with mild-to-moderate CD [3,74]. In addition, EEN therapy has demonstrated an improved nutrition status, growth, mucosal healing, reduced fecal calprotectin, normal CRP remission, and a high safety profile [3,67,75]. The use of EEN for induction of remission does not come with increased use of biologicals or need for surgery but ultimately leads to a long-term avoidance of steroids in up to half of CD patients during their years of growth [21]. A nutrition-based induction strategy avoids steroid-related side-effects, such as growth retardation and risk of infections [76,77]. The efficacy of EEN-induced remission is independent of the formula types or the administration route [78,79]. However, a polymeric formula is preferred in daily practice due to lower costs, superior taste, and better tolerance [80].

EEN is associated with minimal and temporary side-effects. The most commonly reported side-effects are nausea, diarrhea, constipation, abdominal pain, bloating, and taste fatigue [81]. The only severe adverse event that has (rarely) been reported is refeeding syndrome, a potentially fatal metabolic complication that can occur when starting EEN in children with severe malnutrition but can be avoided with careful monitoring of electrolytes [82,83,84]. Although the vast majority of side-effects are minimal, the acceptability of the patient and their family is a major challenge in the use of EEN. The tolerance of EEN is estimated to be around 74%, where most patients will refuse to continue the treatment [3].

EEN has not only been investigated for induction of remission but also for maintaining remission. Belli et al. investigated the effect of intermittent nocturnal EEN during a one-year follow-up in children with CD with growth failure: 8 subjects received and completed EEN for one month, three times in total, with a three-month break in between each EEN regimen. They showed significant height and weight gains, along with a decreased PCDAI score and a decreased need for steroids [85]. Nevertheless, although EEN is the only well-established, evidence-based dietary therapy, with high rates of remission in pediatric CD [9], the use of EEN is not practical or appealing to continue long term. The rigorous requirements to avoid solid food and the resulting disturbance of normal dietary habits make EEN unsuitable as an effective approach to sustain remission [3]. In addition, there is a rapid loss of response and return of inflammation upon food reintroduction in patients after EEN induction therapy [76].

### 4.2. Partial Enteral Nutrition (PEN)

With the well-described beneficial effects of EEN, PEN has also been studied in the hope of creating a more patient-friendly, tolerable therapy to achieve induction or maintain remission [86]. PEN uses the same liquid formulas as EEN but for less than 100% of caloric needs, in addition to some regular daily food intake. The use of PEN, as investigated in clinical studies, varies from 10–90% of calculated caloric daily intake [86,87]. Different studies have shown an improvement of CD symptoms with the use of PEN for inducing remission. However, the effect is usually explained by symptomatic improvements like reduction in abdominal pain and weight gain [86]. Although these are important aspects that contribute to the overall health of the patient, they are not necessarily due to reduced inflammation and improved mucosal healing [86]. Indeed, PEN with a free diet was not able to induce remission and suppress inflammation in active CD [76,86]. It was also significantly less potent compared to EEN and anti-TNF for inducing mucosal healing and decreasing inflammation [88]. However, adherence rates to PEN are usually low, which could partially explain the lack of effect in different studies.

The effect of PEN alone or PEN combined with medical therapy for maintenance of remission has also been shown to be of benefit. These studies have been done mainly in adults [89,90]. Although some studies have shown benefits from the use of PEN for maintenance of remission in pediatric CD, the data remains inconclusive [91,92,93,94]. Short-term PEN as supportive treatment (in addition to regular therapy) for 4 weeks after induction of remission with medical therapy showed an improved nutritional status after 1 year in children with severe CD [94]. Similarly, nocturnal PEN for 4–5 nights/week, in addition to an ad libitum diet, showed prolongation of EEN- and corticosteroid-induced remission and improved linear growth in children and adolescents [91]. In the CERISIER trial conducted in Japan by Hisamatsu et al., PEN combined with an escalation of anti-TNF dosing in secondary loss-of-response was superior to dose-escalation alone [95]. The study was halted early after an intermediate analysis was performed on 15 patients that showed the benefits of combination therapy and the disadvantage of anti-TNF escalation alone. Compared with anti-TNF escalation alone, the combination group showed a tendency toward a superior response rate to infliximab (IFX; 10 mg/kg every 8 weeks), with week 56 as the primary endpoint, but, likely due to the early discontinuation of the study and the consequently reduced number of patients, significant differences at week 56 were not seen. This appears to be the first clinical trial to show the usefulness of combination therapy of enteral nutrition (EN) therapy with biologics for anti-TNF refractory CD [95].

In a recent meta-analysis based on studies in children and adults with CD, Gkikas et al. concluded that the consumption of more than 35% of caloric needs that come from EN is necessary to achieve clinical benefits in maintaining remission [96]. The ESPHGAN guideline recommends a daily caloric intake of at least 50% to reach therapeutic efficacy for effective prolongation of remission in low-risk CD [9]. Recently, different studies have investigated the role of PEN in combination with specific diets such as CDED and anti-inflammatory diet (AID)-CD, reaching promising results and showing the effectiveness of combination therapy with PEN and other specific diets [3,97,98,99].

### 4.3. Crohn’s Disease Exclusion Diet (CDED)

The recently described CDED is a whole-food diet coupled with PEN (*MODULEN*™ *IBD*, Nestlé) [3,99]. CDED is a structured diet designed to reduce exposure to dietary components that may negatively affect the microbiome, intestinal barrier, and intestinal immunity [3,99]. CDED limits exposure to animal fat, certain types of meat, gluten, maltodextrin, emulsifiers, sulfites, and certain monosaccharides [3,99]. CDED was described for the first time in a case series of adults and children with CD by Sigall-Boneh et al. [99]. Participants followed CDED, with additional PEN for 50% of calculated energy requirements for 6 weeks, followed by a step-down diet with an additional 25% of energy requirements by PEN [99]. Patients following CDED + PEN therapy reached high rates of clinical response and remission (around 80%) [99,100]. Interestingly, the majority of patients who refused additional PEN and followed only CDED also reached clinical remission [99]. PEN was initially added to the diet to guarantee the full caloric and nutritionally balanced intake of the subjects. Data from this study, however, indicated that PEN is not necessary to achieve remission [99]. This was confirmed in a recent RCT in adults, where the use of PEN was numerically but not statistically superior in remission induction [101]. However, the use of PEN is still preferred due to its nutritional status benefit and PEN being a major source of calcium during treatment with CDED [94,102].

**Table 1:** Summary of the nutritional therapy intervention studies in children with Crohn’s disease, as described in Section 4.

Ref	Study Design	Aim	Population (Activity)	Intervention (Duration), *n*	Comparator (Duration), *n*	Key Findings
***Exclusive Enteral Nutrition***						
Belli et al. [85]	Prospective	To reestablish growth	Pediatric CD with growth failure *(n.r.)*	Intermittent EEN (1 year: 3 times 1 month EEN, with 3 month breaks) *n* = 8	No EEN, matched *n* = 4	-Intermittent EEN show sign. decrease in CDAI and prednisone intake -Intermittent EEN shows sign. height and weight gain -Similar rate of pubertal development in both groups
Ludvigsson et al. [78]	RCT	To compare efficacy and safety of elemental and polymeric diets	Pediatric CD *(PCDAI > 12)*	Elemental formula (6 weeks) *n* = 17	Polymeric formula (6 weeks) *n* = 18	-Similar remission rates in both groups at week 6 -Sign. higher weight gain in polymeric formula
Rubio et al. [79]	Retrospective	To analyze the efficiency of oral fractionated versus continuous enteral feeding	Pediatric CD *(n.r.)*	EEN oral (8 weeks) *n* = 45	EEN continuous enteral (8 weeks) *n* = 61	-Similar remission rates in both groups -Similar effects on PCDAI and inflammatory markers in both groups -Sign. more weight gain in enteral feeding group
Connors et al. [21]	Retrospective, propensity score-matched	To compare short- and long-term disease outcomes	Pediatric new-onset CD *(PCDAI ≥ 10)*	EEN (8–16 weeks) *n* = 82	CS (n.r.) *n* = 45	-EEN sign. more effective in inducing remission -EEN associated with long term steroid avoidance over 6 years -Similar outcomes on long term linear growth, hospitalization, need for biologic therapy and surgical intervention in both groups
Pigneur et al. [72]	RCT	To study anti-inflammatory effects and its modulatory effect on the microbiota	Pediatric new-onset CD *(HBI > 5)*	EEN (8 weeks) *n* = 13	CS (4 weeks, tapered) *n* = 6	-EEN induces sign. higher mucosal healing at week 8 -Similar drop in inflammatory markers at week 8 -Higher proportion of Ruminococcus bacteria and bacteria belonging to clostridium genus in EEN group
Logan et al. [76]	Observational	To study changes in FCP during EEN and at food reintroduction and explore associations with MEN	Pediatric CD *(n.r.)*	EEN (8 weeks) *n* = 68 optional MEN	-	-Sign. increase in FCP within 17 and 52 days after food reintroduction -Sign. lower FCP in MEN group compared to EEN at day 17 -MEN not associated with prolonged remission
***Partial Enteral Nutrition***						
Wilschanski et al. [91]	Retrospective	To examine whether continuation of EN as nocturnal supplement lengthens remission	Pediatric CD successfully treated with EEN *(n.r.)*	PEN nocturnal (n.r.) *n* = 28	No PEN *n* = 19	-Additional PEN induces sign. prolongation of remission at 6 and 12 months -Additional PEN induces sign. increased linear growth
Johnson et al. [86]	RCT	To compare PEN with EEN for induction of remission	Pediatric CD *PCDAI > 20*	PEN (6 weeks) *n* = 26	EEN (6 weeks) *n* = 24	-EEN sign. superior to PEN in inducing clinical remission -EEN and PEN both induce sign. drop in PCDAI -PEN PCDAI drop due to/symptomatic/nutritional benefits -EEN suppresses inflammation
Kang et al. [94]	Prospective open-label study	To examine the effects of concomitant use of PEN as adjuvant therapy	Pediatric severe CD *PCDAI > 45*	PEN (4 weeks) *n* = 18	Normal diet *n* = 16	-PEN improved nutritional status sign. after 1 year
Lee et al. [88]	Prospective	To compare effectiveness between PEN, EEN, and anti-TNF therapy for induction of remission	Pediatric CD *PCDAI > 10*	EEN (8 weeks) *n* = 22 PEN (8 weeks) *n* = 16	Anti-TNF (n.r.) *n* = 52	-Clinical remission PEN 50%, EEN 76%, anti-TNF 73% -EEN sign. superior in inducing remission -FCP < 250 ug/g sign. higher in EEN and anti-TNF -Improvement QOL similar in all groups
Schulman et al. [92]	Retrospective	To evaluate efficacy of PEN for preventing clinical relapse	Pediatric CD successfully treated with EEN *(n.r.)*	PEN as supplementary diet (median 6 months) *n* = 42	No PEN *n* = 45	-Similar duration of maintenance of remission in both groups -Suppl. PEN sign. increases weight and BMI
***Specific diet***						
Sigall et al. [99]	Retrospective	To report on experience with CDED and its efficiency in inducing remission	Pediatric and young adults with CD *PCDAI ≥ 10* *HBI > 3*	CDED + PEN (12 weeks) *n* = 40 CDED alone (12 weeks) *n* = 7	-	-Remission achieved in 78.8% of participants at week 6 (24/34 children and 9/13 adults) -Remission maintained in 84% of participants at week 12 -Remission in 6/7 participants on CDED alone -Sign. reduction of PCDAI and inflammatory markers
Sigall et al. [98]	Retrospective	To report on experience of CDED for induction of remission in patients with loss of response to infliximab/adalimumab or combination therapy	Pediatric and young adults with CD with flare/active inflammation on biological *(n.r.)*	CDED + PEN (12 weeks) *n* = 12 CDED alone (12 weeks) *n* = 4 Modified EEN + CDED (2 + 12 weeks) *n* = 5	-	-Clinical remission 62% (13/21) -Among patients failing combination therapy 53% reach remission (9/17) -Sign. reduction of inflammatory markers overall
Levine et al. [3]	RCT	To study tolerability of the diet and efficacy in induction of remission	Pediatric mild to moderate CD *10 ≤ PCDAI ≤ 40*	CDED + PEN (12 weeks) *n* = 40	EEN (6 weeks) *n* = 34	- CDED and EEN equally effective in inducing remission at week 6 - CDED superior in sustained remission at week 12 -CDED sustained changes in fecal microbiome at week 12 -CDED and EEN induce a rapid clinical response (by week 3) -Identification of patients with a rapid response to diet could identify those who will be in clinical remission by week 6 with good compliance [103]
Svolos et al. [44]	Open-label	To test efficacy of CD-TREAT to induce clinical remission and ameliorate inflammatory markers	Pediatric relapsing CD *wPCDAI ≥ 12.5*	CD-TREAT (8 weeks) *n* = 5	-	-CD-TREAT induced clinical response 80% (4/5) -CD-TREAT induced clinical remission 60% (3/5) -CD-TREAT induced decrease in fecal calprotectin 4/5
Urlep et al. [97]	RCT	To compare clinical and endoscopic remission rates and mucosal healing	Pediatric CD *PCDAI > 10*	AID-CD + PEN (6 weeks) *n* = 12	EEN (6 weeks) *n* = 13	-AID-CD and EEN equally effective in inducing clinical and endoscopic remission -EEN had superior mucosal healing, although not significant

RCT: randomized controlled trial. CD: Crohn’s disease. EEN: exclusive enteral nutrition. CS: corticosteroids. HBI: Harvey Bradshaw Index. PCDAI: Pediatric Crohn’s Disease Activity Index. FCP: fecal calprotectin. MEN: maintenance enteral nutrition. EN: enteral nutrition PEN: partial enteral nutrition. CDED: Crohn’s disease exclusion diet. CD-TREAT: CD treatment with diet. AID-CD: anti-inflammatory diet–CD; sign.: significant. *n*: sample size. n.r.: not reported.

The efficacy of CDED + PEN compared to EEN in inducing clinical remission in children with mild-to-moderate CD was recently shown in a multinational RCT [3]. CDED + PEN and EEN showed equal effectiveness in inducing remission at week 6, with remission rates around 85%. In addition, 80% of children with a response to EEN or CDED + PEN at week 3 reached clinical remission at week 6 [103]. However, CDED + PEN demonstrated better tolerance by children and their parents at week 6, addressing the adherence challenges of EEN. One out of 40 patients in the CDED + PEN group withdrew due to low compliance or intolerance to the diet. In the EEN group, 7 out 34 children discontinued, of which 6 refused to continue EEN. In addition, CDED + PEN led to better-sustained remission through to week 12, which was achieved in 75% of the children [3,100]. Importantly, CDED + PEN sustained remission was associated with sustained microbiome changes associated with remission [3,104]. In previous studies performed on both children and adults with CD, including patients with secondary loss of response to biologic therapy [98,99], combining CDED with immunomodulators often led to successful recapturing of remission in these patients. These results are promising as this specific group is in urgent need of alternative therapeutic options.

CDED shows promising therapeutic potential for pediatric CD, not only for induction of remission but also as maintenance therapy, drug de-escalation, or rescue therapy in children with loss of response to other treatments [100]. However, following the CDED also requires parental commitment to the planning and preparation of meals according to the dietary instructions. In addition, studies and data of its effects on mucosal healing and inflammation, as stated in the recently published ESPHGAN guideline, are eagerly awaited [9].

### 4.4. CD Treatment-with-Eating Diet (CD-TREAT)

The individualized food-based diet CD-TREAT is an ordinary (solid) food diet that recreates the composition of EEN (*MODULEN*™ *IBD*, Nestlé), excluding dietary components like gluten and lactose and matching others like carbohydrates and proteins [44]. The therapy aims to mimic EEN’s effects on the gut microbiome, metabolome, inflammation, and clinical outcomes [44]. In an RCT performed on 25 healthy volunteers (adults) that received EEN and CD-TREAT for 7 days, each with a 14-day washout period in between, the effects of CD-TREAT were comparable to EEN in terms of microbiome changes and the composition of a range of metabolites [44]. Many of these effects observed in human volunteers were confirmed in an animal model [44]. Additionally, in a pilot of 5 children with active CD, CD-TREAT administration for 8 weeks showed the benefit of inducing clinical remission and a reduction of fecal calprotectin [44]. There was high adherence to the diet during the study, which did arrange for meals to be prepared and distributed for free by a local provider to participants [44]. Although these results are promising, they remain to be confirmed in an ongoing and sufficiently powered RCT [105].

### 4.5. Recommendations for Clinical Practice

New ESPHGAN-ECCO guidelines recommend EEN as the first choice of therapy for the induction of remission for mild-to-moderate luminal CD [9]. CDED + PEN can be considered a better-tolerated alternative with a similar rate of induction of remission by 6 weeks and improved maintenance of remission at 12 weeks [3]. PEN alone is not recommended for induction of remission but can be used to prolong remission or as a short-term bridge between therapies [9]. Other dietary strategies will need further research before incorporation into daily clinical practice can be recommended.

## 5. Potential Mechanisms of Action of Nutritional-Therapy-Induced Remission

**Figure 1:**
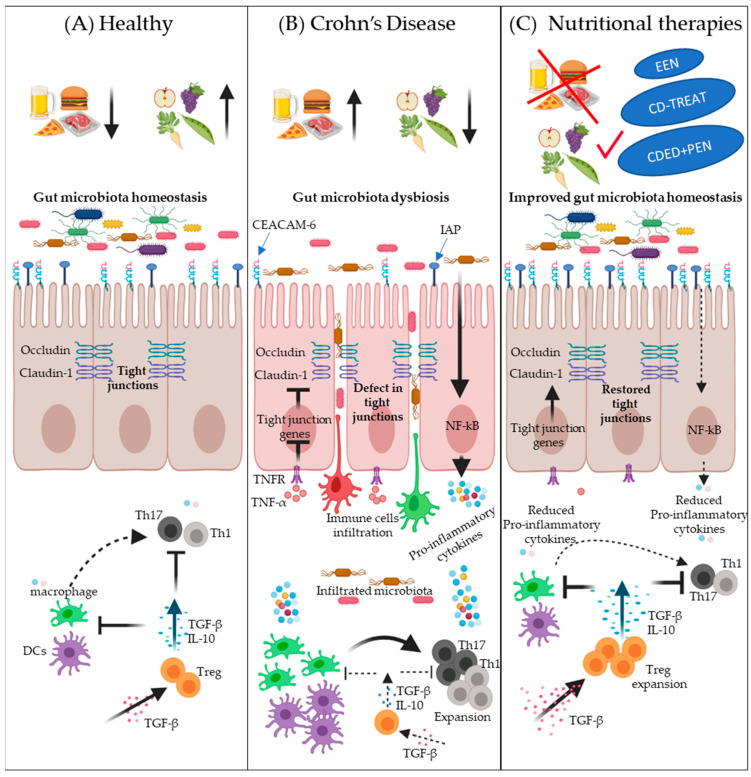
Schematic overview of some potential mechanisms of nutritional-therapy-induced remission in Crohn’s disease (CD). Panel (**A**) depicts the intestinal homeostasis in health associated with a healthy diet, i.e., less intake of a Westernized diet (normal microbiota diversity, normal expression of IAP and CEACAM-6, less interactions between bacteria and epithelial cells, normal expression of tight junction proteins such as occludin and claudin-1, less penetration of epithelium by potentially pathogenic bacteria and infiltration of immune cells, better immune surveillance by Tregs, and normal expression of TGF-β). Panel (**B**) illustrates the potential effects of a Westernized diet on the development of CD: low expression of antigen-related cell adhesion molecule (CEACAM-6) and intestinal alkaline phosphatase (IAP), decreased tight junction proteins expression, defects in intestinal barrier function, increased bacterial adherence to epithelial cells, stimulation of epithelial cells to express proinflammatory cytokines, increased bacterial and immune cells infiltration, reduced TGF-β, decreased Treg cells expansion, increased expansion of proinflammatory T-cells, and elevated proinflammatory cytokines expression by immune cells. Panel (**C**) describes the potential of nutritional therapies to correct dysbiosis and to reduce intestinal inflammation. Nutritional therapies can enhance microbial diversity and increase the expression of CEACAM-6 and IAP to prevent the adherence of bacteria with epithelial cells. Nutritional therapies can also reduce proinflammatory-cytokine-induced barrier dysfunction, such as TNF-α, thus normalizing the expression of tight junction proteins and improving intestinal barrier function, which, in turn, leads to less infiltration of microbes and immune cells. TGF-β supplementation has a beneficial effect on T-cell polarization into a Treg phenotype, which inhibits T-cell polarization into Th1 and Th17 subsets as well as dampening inflammatory macrophage function.

Nutritional therapies have shown a great ability to attenuate intestinal inflammation and induce mucosal healing in pediatric CD patients [3,44,71,99]. However, the mechanisms by which they induce and sustain remission remain unclear [106]. The enhancement in nutritional status, promoting an anti-inflammatory response and increasing the production of innate defense proteins, restraining luminal antigen exposure, improving gut permeability, and changes in gut microbiota have been suggested as potential mechanisms [107]. Some of the potential mechanisms of action of nutritional-therapy-induced remission are illustrated in Figure 1.

### 5.1. The Direct Anti-Inflammatory Potential

Nutritional therapies such as CDED and EEN have been shown to reduce intestinal inflammation in pediatric CD, reflected by inflammatory markers such as CRP and calprotectin [3]. Several studies have shown the direct anti-inflammatory properties of nutritional therapy [106]. The first evidence in this direction was reported by Logan et al., Breese et al., and Beattie et al. [56,108,109]. They showed that EEN reduces the frequency of cytokine-producing cells in the mucosa of CD patients [56,108,109]. In addition, gene and protein expression levels of intestinal proinflammatory cytokines, including IL-1β, IFN-γ, and TNF-α, declined in CD patients after a few weeks of the EEN polymeric diet or the EEN elemental diet [56,57,58]. This observation was confirmed in vitro [110]. A polymeric formula used in EEN was able to downregulate the expression of cytokine genes downstream of the NF-κB pathway, such as *IL8* and *IL6* in HT-29 colonic epithelial cells in response to TNF-α [110]. The essential amino acid components of the polymeric formula, glutamine and arginine, were responsible for this effect by disrupting the phosphorylation of cascade elements in NF-κB and P38 signaling and downregulating kinase activity [111]. Peripheral blood mononuclear cells (PBMCs) isolated from children with CD during EEN and incubated with LPS or flagellin exhibited lower secretion of proinflammatory cytokines such as IL-6, IL-8, IL-1b, and IFN-γ [59]. Notably, EEN enhances the ability of IL-10 to suppress the inflammatory response of those PBMCs [59].

Alterations in microRNAs have also been suggested to mediate anti-inflammatory effects in CD patients during nutritional therapies [112]. Microarray analysis of mucosa from patients that were subjected to EEN therapy showed a clear normalization of many IBD-associated microRNAs [113]. This suggests that EEN may play a role in the post-transcriptional regulation of gene expression. As EEN is typically very low in fiber, EEN has been shown to reduce fecal levels of SCFAs such as butyrate [114]. Butyrate regulates the expression of epigenetic enzymes, notably, histone deacetylases (HDACs) that have a role in controlling the immune response and gut activity [115,116].

Intestinal alkaline phosphatase (IAP) and antigen-related cell adhesion molecule (CEACAM)-6 are involved in the innate intestinal immune response against enteric pathogens, preventing bacterial interactions with epithelial cells [117,118]. Adherence of bacteria to and penetration within epithelial cells and their interaction with dendritic cells and macrophages leads to ongoing activation of the mucosal immune system [117,118]. Adding a polymeric formula to epithelial cell lines increased IAP and CEACAM-6 [117,118].

Transforming growth factor-β (TGF-β) is an immune-suppressive cytokine; disrupted TGF-β signaling has been detected in the intestines of IBD patients [119,120]. TGF-β favors the polarization of T-cells into T regulatory cells (Treg) [121]. It was observed that EEN therapy enhances TGF-β1 in blood [61,122], suggesting that EEN may direct the polarization of T-cells into the Treg phenotype, which, in turn, reduces proinflammatory T-cells in CD. TGF-β2 has been added to EEN formulas, which may have the additional effect of reducing intestinal inflammation [16,61,123,124,125].

Proinflammatory T-cells such as Th1 and Th17 are critical in promoting inflammation in CD [126]. Different T-cell phenotype subsets were assessed in PBMCs isolated from children with CD that have been treated with EEN. EEN increased FOXP3^+^ Tregs and reduced Th1-derived IFN-γ [59]. No changes in the frequency of the Th2 phenotype were seen [59]. Further work is required to convincingly understand these processes.

### 5.2. The Improvement of Intestinal Barrier Function

The intestinal inflammation observed in active CD is accompanied by increased intestinal permeability, disrupted integrity of the mucus layer, and lower barrier effectivity against bacteria and proinflammatory mediators [16]. Accumulating evidence suggests that dietary products play a role in impairing intestinal barrier function in CD [3,45]. Nutritional therapies improve mucus integrity and reduce permeability in pediatric CD [21,67,74]. A possible mechanism is that nutritional therapies allow the reestablishment of a functional epithelial barrier by excluding foods (including certain additives) that may adversely affect barrier function [127]. In an in-vitro model of TNF-α induced barrier dysfunction in Caco-2 monolayers, adding a polymeric formula improved barrier integrity [128]. The polymeric formula prevents the alterations of some tight junction proteins such as occludin, claudin-1, and ZO-1 [128], which were also seen in a murine colitis model subjected to EEN [129]. This model showed that these corrections of intestinal tight junctions can be mediated by downregulating the myosin light-chain kinase [129]. Specific components of EEN, such as glutamine, have been implicated in reducing permeability and improving barrier integrity [130]. By lowering proinflammatory cytokines, nutritional therapy can reduce, e.g., TNF-α-induced barrier dysfunction [110].

### 5.3. Microbiome Dysbiosis Correction

Intestinal microbiota is essential in maintaining homeostasis; the link between altered diet, dysbiosis, and induction of inflammation in CD is well established [131,132]. Nutritional therapies have shown great potential in correcting CD dysbiosis [3,25,44]. Earlier studies have used gradient gel electrophoresis (GGE) to demonstrate the effect of nutritional therapies on gut microbiota [133,134,135]. Leach ST et al. have shown that EEN induces a significant and prolonged decrease in bacterial diversity in the feces of children with CD, especially in the group of *Bacteroides*. These changes were associated with a marked reduction of inflammation [133].

16S rRNA sequencing and whole-genome sequencing have revolutionized the study of the association between diet and microbiota changes [3,133,136]. In a prospective clinical study on pediatric CD patients, 16S rRNA sequencing reported marked changes in fecal bacterial communities after 2 weeks of EEN, in particular, the reduction in Gram-negative bacteria abundance that belongs to the *Bacteroidetes* phylum (including *Bacteroidaceae*, *Porphyromonadaceae,* and *Rikenellaceae*) and the increase in Gram-positive bacteria belonging to the *Firmicutes* phylum (especially the *Christensenellaceae* family) [59]. An EEN-induced increase in *Bacteroidetes* was also reported in other studies [114,133,137]. In healthy conditions, around 90% of bacterial gut species are part of the *Firmicutes* and *Bacteroidetes* phyla [138]. However, the dysbiosis associated with CD is generally characterized by a reduction in *Firmicutes* and an expansion of *Proteobacteria*, as well as an overall decrease in bacterial diversity [139,140].

Changes in fecal microbial composition induced by EEN or CDED + PEN have been linked with remission and sustained remission [3,100,141,142]. In an RCT, both CDED + PEN and EEN were effective in inducing remission by week 6 and showed similar taxa changes: a decrease in *Actinobacteria* and *Proteobacteria* and an increase in *Clostridia* [3]. CDED + PEN was able to sustain remission through to week 12, which was associated with increased *Actinobactera* and a sustained decrease in *Proteobacteria,* while EEN was not effective in sustaining clinical remission until week 12 and demonstrated a major rebound in *Proteobacteria* [3].

Multiple studies have described the paradoxical loss in diversity upon the start of EEN therapy in children with CD, moving even further away from healthy controls in terms of the metrics of microbiome diversity. This diversity effect reverses upon return to the free diet [114,142,143,144]. However, diversity is a poor measure of microbiome complexity, as patients who do not achieve or sustain remission have been shown to expand *Proteobacteria* and lower-species-richness (using amplicon-sequence variant analysis), which are associated with lower calprotectin [25,137].

Bile acids (BAs) play a principal role in regulating immune responses and bacterial community composition within the gut; changes in intestinal microbial communities can disturb intestinal BA composition [142,145,146]. Altered BA metabolism has been demonstrated in the feces of CD patients and is associated with decreased secondary BAs (secBAs) and increased primary BAs (priBAs) compared to healthy controls [147,148]. Connors et al. have investigated the effect of EEN-therapy-induced remission associated with changes in fecal microbiota on BA profiles in children with CD [145]. priBAs were predominant in samples that did not achieve or sustain remission with EEN [145]. However, patients who achieved and sustained remission with EEN had predominantly secBAs. Notably, different microbial communities were detected in priBAs vs. secBA-dominant samples [145]. While priBA-dominant samples exhibited mainly *Bacteroides* (including *B. plebeius*), *Enterobacteriaceae, Roseburia, Ruminococcus gnavus,* and *Megamonas*, secBA-dominant samples contained predominantly different groups of *Bacteroides* (including *B. uniformis*), *Ruminococcaceae, Erysipelotrichaceae, Rikenellaceae,* and *Lachnospiraceae* [145].

Further studies are warranted to investigate the effect of nutritional therapies on a range of metabolic pathways involved in IBD, such as tryptophan [149,150], sphingolipid [151], and purine [152] metabolism, as data do not suggest SCFA production and BA composition to be the key pathways involved in achieving diet-induced remission [114,153,154].

## 6. Conclusions

There has been an increasing interest in the potential of nutritional therapy strategies in different diseases, including CD. Dietary patterns are associated with an increased risk of developing CD, and their modification can be harnessed to influence disease course. There is considerable preclinical evidence showing the potential mechanisms of different dietary factors on inflammation, intestinal barrier function, and the gut microbiome. The effect of EEN on children with CD has directed attention towards the development of new nutritional treatment strategies. Nutritional therapy by dietary modulation, using whole-food diets such as the CDED, has shown improved tolerability with comparable effectiveness to EEN. More research is needed to position dietary modification, together with medical therapy, for both the induction and maintenance of remission. 

## Data Availability

Data sharing not applicable. No new data were created or analyzed in this study. Data sharing is not applicable to this article.
